# Current Trends in Incidence and Management of T1a and T1b Prostate Cancer

**DOI:** 10.7759/cureus.40224

**Published:** 2023-06-10

**Authors:** Firaas A Khan, Ahmad Imam, David J Hernandez

**Affiliations:** 1 Department of Medicine, University of South Florida Health - Morsani College of Medicine, Tampa, USA; 2 Department of Urology, University of South Florida, Tampa, USA

**Keywords:** holep, prostate cancer, t1b, t1a, ipca

## Abstract

Prostate cancer (PCa) identified incidentally (iPCa) after surgical treatment for symptomatic benign prostatic hyperplasia (BPH) causing lower urinary tract symptoms (LUTS) is considered low risk by the most current guidelines. Management protocols for iPCa are conservative and are identical to other prostate cancers classified as having favorable prognoses. The objectives of this paper are to discuss the incidence of iPCa stratified by BPH procedure, to highlight predictors of cancer progression, and to propose potential modifications to mainstream guidelines for the optimal management of iPCa. The correlation between the rate of iPCa detection and the method of BPH surgery is not clearly defined. Old age, small prostate volume, and high pre-operative prostate-specific antigen (PSA) are associated with an increased likelihood of detecting iPCa. PSA and tumor grade are strong predictors of cancer progression and can be used along with magnetic resonance imaging (MRI) and potential confirmatory biopsies to determine disease management. In instances that iPCa requires treatment, radical prostatectomy (RP), radiation therapy, and androgen deprivation therapy all have oncologic benefits but may be associated with increased risk after the BPH surgery. It is advised that patients with low to favorable intermediate-risk prostate cancer undergo post-operative PSA measurement and prostate MRI imaging before electing to choose between observation, surveillance without confirmatory biopsy, immediate confirmatory biopsy, or active treatment. Subdividing the binary T1a/b cancer staging into more categories with ranging percentages of malignant tissue would be a helpful first step in tailoring the management of iPCa.

## Introduction and background

Prostate cancer (PCa) is the most frequent malignancy type and the second-most common cause of cancer-related deaths in men [1] that often arises with concomitant benign prostatic hyperplasia (BPH) in approximately 83% of cases [2]. In rare cases, PCa is identified incidentally (iPCa) after surgical treatment of lower urinary tract symptoms (LUTS) in patients with BPH. IPCa is clinically imperceptible and is discovered upon microscopic examination of resected tissue after BPH surgery [3]. These cancers are currently classified as either T1a or T1b where cancer is found in less than or more than 5% of resected tissue, respectively. While the gold standard for BPH treatment is transurethral resection of the prostate (TURP), there are many alternative options available including simple prostatectomy (SP), holmium laser enucleation of the prostate (HoLEP), and more [4].

According to the American Urological Association (AUA), National Comprehensive Cancer Network (NCCN), and European Association of Urology (EAU) guidelines, iPCa is considered low-risk, and current treatment recommendations are not unique to other low-risk PCas [5-7]. Generally, these guidelines advocate that patients with low-risk PCa be managed conservatively with active surveillance (AS) which entails serial prostate-specific antigen (PSA) values and repeat prostate biopsies. Although it seems reasonable for iPCa to be treated similarly, due to the low probability that it would grow or progress to invade other structures, the question arises of whether this is optimal considering the uncommon diagnostic course compared to other PCas with favorable prognoses. Additionally, as the worldwide population and the number of BPH surgeries continue to increase, like in Australia where Patel and Bariol reported a 79% increase in BPH surgeries from 2008 to 2017, there is concern that the current classifications for iPCa are antiquated and that the concurrent recommendations are inadequate [8]. This paper aimed to discuss the current literature relevant to the incidence of iPCa, important predictors of disease progression, and feasible suggestions that could improve iPCa management.

## Review

Methods

A comprehensive literature review was conducted using the PubMed and Google Scholar databases to identify relevant studies pertaining to the investigation of incidental prostate cancer (iPCa) rates, trends of iPCa progression, and the management of iPCa. Specific search strings were formulated using a combination of relevant keywords, such as "incidental prostate cancer," "iPCa," "management," "incidence," "biochemical recurrence," "survival," and "complications." Studies were included if they met the following criteria: (1) they were published in peer-reviewed journals, (2) they investigated iPCa rates, trends of iPCa progression, and/or the management of iPCa, (3) they were written in English, (4) they were published between 1980 and 2023, and (5) they were available in full text. The extracted data were analyzed to provide a comprehensive overview of the rates, progression, and management strategies for iPCa.

Predicting iPCa in common surgeries for BPH

The rate of iPCa detection in common procedures, such as TURP, SP, and HoLEP varies in the reported literature. According to a systemic review conducted by Cheng et al. in 2022, the historical incidence of iPCa in TURP procedures ranged from 1.4% in some studies to 16.7% in others [9]. The incidence of iPCa in HoLEP studies was also quite variable, ranging from 2.5% to 23%. Over the years, there has been an overall decrease in the detection of iPCa due to the introduction of PSA screening tools in the early 1990s [10].

Although procedures, such as HoLEP and SP are known to involve the removal of a greater amount of prostate tissue, the association between the extent of tissue removal and the detection of iPCa remains inconclusive. For example, a study by Herlemann et al. in 2017 compared the likelihood of discovering cancer in patients that underwent HoLEP vs. TURP, and they found no significant difference in the iPCa detection rates between the groups (15% for HoLEP vs. 17% for TURP, p=0.593) [11]. They concluded that the choice of technique has no influence on the diagnosis of cancer. Another study found an increased removal of prostatic tissue in HoLEP compared to other BPH procedures, but this did not impact the diagnosis of iPCa [12]. Other studies, such as He et al. in 2020 also supported the fact that HoLEP removed more tissue from the prostate but also; however, they found that there was an increase in the detection of iPCa for HoLEP than for TURP [13]. The detection rate of iPCa was 6.24% for HoLEP group and 3.94% for TURP group (p=0.005). Rosenhammer et al. in 2018 displayed similar findings using a case-by-case matched pair-analysis and showed HoLEP is advantageous in detecting iPCa compared to TURP due to the higher percentage of tissue removed and superior preservation of tissue adjacent to the peripheral zone [14]. IPCa was found in 23.3% of HoLEP patients compared to 8.3% in TURP (p=0.043). In the PSA era, incidental prostate cancer (iPCa) without prior diagnosis has been found in TURP specimens in 5% to 13% of the patients [15]. Other studies have shown no difference in iPCa rates between SP and HoLEP [16] as well as SP and TURP [17]. Capogrosso et al. in 2018 reported a higher iPCa rate in HoLEP patients; however, they cautioned that this value may be skewed as a result of a greater number of HoLEP procedures performed in recent years [17]. Overall, clear differences in the efficacy of detecting iPCa between these different surgical methods cannot be fully established.

Various factors are described as being linked to iPCa. Older age is a risk factor for iPCa with patients >80 years of age having a 20% predicted probability of diagnosis [18]. Sakamoto et al. in 2014 also described that smaller prostate volume and the absence of previous needle biopsy (despite a high PSA level) are significant risk factors for detecting iPCa [15]. In a study by Otsubo et al. in 2015, low prostate volume, high PSA, high PSAD, and high PSA velocity were all significantly associated with iPCa discovery in patients who underwent HoLEP [19]. Interestingly, these patients also tended to have a lower weight of removed prostatic tissue than those with only BPH.

The use of PSA testing in screening for prostate cancer (PCa) is often a subject of debate due to its lack of specificity, as elevated levels can be found in individuals without prostate cancer, leading to false alarms and subsequent patient anxiety. Furthermore, the cutoff values for defining abnormal PSA levels are still a matter of ongoing debate, with different guidelines offering conflicting recommendations. This inconsistency can result in varying interpretations and management decisions. Moreover, PSA screening may not effectively differentiate aggressive cancers from indolent ones, potentially leading to both overtreatment and undertreatment. However, it is important to note that prior to the introduction and widespread use of PSA screening, incidental prostate cancer (iPCa) was found in approximately 30% of transurethral resection of the prostate (TURP) specimens [20]. Despite the challenges in interpreting PSA testing, it has demonstrated benefits in reducing the incidence of iPCa.

Notably, water vaporization methods of treating BPH, including Rezum and Aquablation, have risen in popularity; however, they do not allow for subsequent histological examination [21]. Therefore, patients that undergo these procedures should be counseled about the risk of missing iPCa.

Indicators of cancer progression

Gleason grade scores which guide tumor grading and PSA levels are typically used as indicative markers for determining whether or not a patient with PCa would benefit from invasive forms of treatment; however, when or whether to treat iPCa is still a challenging dilemma [22,23]. It is classically regarded, for example, that T1a cancers should be monitored through active surveillance or watchful waiting while T1b cancers should be treated aggressively as there is a higher chance of disease progression as shown in studies such as Cantrell et al. in 1981 which found a higher risk of progression for T1b compared to T1a (29% vs. 8%; after 17 months vs. 73 months) [24]. Other more recent studies have since contended this point, such as Capitanio et al. in 2008 concluded that the T1a vs T1b stage distinction did not accurately predict residual cancer or biochemical recurrence [22]. High levels of PSA and prostate-specific antigen density (PSAD) have also proven to be effective markers for predicting iPCa incidence [25] and progression [22]. After the introduction of PSA in the late 1980s, the incidence of T1a/b prostate cancers decreased significantly, and in one study of 1648 patients, there was a decrease from 23% to 7% with a greater effect on T1b cancers [26]. Other PSA kinetics values, such as PSA velocity and prostate-specific antigen doubling time (PSADT) have also been shown to predict the progression of iPCa [27].

An interesting topic of debate is the current staging classifications of iPCa which divide patients into two broad categories of tumor infiltration. Many claim this restricts the potential of offering more refined treatment and management recommendations to patients. Multiple studies have corroborated that the percentage of prostatic tissue chips infiltrated by cancer is predictive of survival. One study found that by subdividing patients with T1 cancer into groups of ranging percentages of infiltrative cancerous tissue (≤10%, >10 to ≤25%, >25 to ≤75%, and >75), it offered increased value and improved prediction compared to the current binary division and should be used along with other indicators of disease progression (i.e., PSA and Gleason grade) like grade in predicting cancer-related death [28].

Management trends of iPCa

Current guidelines by the AUA, NCCN, and EAU emphasize the utilization of risk stratification [5-7]. Although the stage is a significant component of risk stratification, PSA and grade groups play an equally important role. Prostate cancer diagnosed via an outlet procedure would be currently classified as T1a/b disease. If this were the only risk stratification tool, then patients would be classified as low risk and active surveillance vs. watchful waiting would be recommended. However, the incorporation of PSA and grade groups could potentially upstage the risk category. In the era of PSA testing, it is pertinent to examine the occurrence of grade group >2 or PSA >10 in patients diagnosed with iPCa. Notably, the incidence of such cases in this patient population is remarkably low. For example, Liu et al. in 2019 described that although 11.2% of 771 patients who underwent TURP had incidentally found prostate cancer, 1% of all cases had GG2 or higher prostate cancer [29]. All patients in this study were managed with watchful waiting and only five patients had PSA progression requiring intervention via radical prostatectomy (RP) or radiotherapy. Additionally, incorporating the use of MRI imaging of the prostate is incredibly useful, along with the other previously mentioned risk stratification tools, such as PSA and tumor grade, in determining what direction should be taken in monitoring and treating iPCa. In a retrospective study on T1a/b cancer patients that had RP, researchers recommended that patients that had both a PSA density of ≤0.08 after BPH surgery as well as invisible cancer lesion with no visible lesions on MRI be considered for active surveillance [30].

Other studies have corroborated these findings. Elkoushy et al. in 2015 performed a review of 1242 patients who underwent HoLEP with the incidence of prostate cancer being 5.64% [25]. Of those with incidental prostate cancer, only 11.7% underwent curative therapy. Tominaga et al. in 2019 recorded the incidence of prostate cancer after HoLEP to be 25 out of 418 (6%) [31]. Only five patients underwent either radiotherapy, RP, or hormone therapy. Two of the five underwent delayed therapy after PSA rise during a period of watchful waiting. A retrospective study conducted by Lee et al. in 2014 identified 156 cases of iPCA after TURP [32]. A total of 29.5% of these patients chose to undergo active surveillance, 42.9% underwent RP, and 21.8% received hormonal therapy. Only two patients in the RP group and three patients in the hormonal therapy group developed recurrence. A study conducted in a single institution in Switzerland spanning from 1999 to 2020 found that active surveillance is a safe option for patients with iPCa following TURP as these patients rarely present with disease progression. Among the few that did progress, the reclassification rate in T1a/b patients was 11.2 years which is nearly three times longer than the reclassification rate in T1c patients at 3.6 years. They concluded that T1a/b patients tend to live longer than T1c patients and that they could be considered for watchful waiting instead of active treatment in many cases [33]. Most recently, Klein et al. in 2022 reviewed the management of iPCa with an incident rate of 10.7% in a large retrospective cohort [3]. Interestingly, in this cohort, there was a much higher risk of progression of those diagnosed with iPCa at a rate of 18.6%. The authors found that a higher risk of progression was associated with T1b disease and PSA ≥2 post-operatively. Among the 25 patients that had disease progression, 68% underwent a combination of external beam radiation therapy and hormonal treatment. Among these patients, only one had recurrence after three years of treatment. Notably among these studies, the incidence of metastatic disease or mortality from prostate cancer was zero. 

Only a few studies have specifically addressed the efficacy and suitability of radiation therapy and RP in patients with iPCa. Though RP after TURP has oncologic benefits, studies suggest it is associated with high complications including urinary incontinence and erectile dysfunction. According to a study conducted by Menard et al. in 2008, patients that underwent RP after TURP had longer operative times, extended hospital stays, and higher positive margin rates [34]. In the study by Jaffe et al. in 2007, patients with a history of TURP were also shown to have worse outcomes with respect to positive margin rate and overall complication rate after RP [35]. Few studies describe RP in patients that have a history of HoLEP. A study from the University of Indiana found that complication rates did not differ between cohorts that did and did not have a history of HoLEP; however, functional recovery was slowed in the former group [36]. Another study looking at the effects of nerve-sparing radical retropubic prostatectomy found that in their small cohort of patients, there was no significant increase in erectile dysfunction among patients that had a history of HoLEP [37].

Radiotherapy has proven to be an appropriate treatment modality for iPCa, and the two main types are external beam radiotherapy (EBRT) and brachytherapy (BT). EBRT can be used for any stage of prostate cancer, while brachytherapy is typically performed for low-intermediate-stage cases. In EBRT, radiation beams are focused on the tumor to deliver a prescribed dose that destroys cancerous cells while sparing normal tissue. Brachytherapy involves placing radioactive seeds or radiation sources either permanently or temporarily in or close to the tumor [38]. These treatments are associated with the potential development of adverse effects like urinary toxicity and urinary incontinence. Although there is varying evidence on the extent of immediate urinary toxicity in patients that underwent radiation therapy and have a prior history of TURP in comparison to those that do not, most studies conclude that this toxicity does not persist [39,40]. In addition, the effect of radiotherapy on urinary continence is minimal, and in one study by Lee et al. in 1996, late urinary incontinence occurs in 2% vs. 0.2% of patients after RT with or without a history of TURP, respectively [41]. There is an increased risk of bladder neck contracture and membranous urethral stricture after radiation therapy in patients with prior outlet procedures, though the incidence of these complications is poorly reflected in the literature.

There are few studies evaluating the incidence of prostate cancer after SP. A case reported by Tsui et al. in 2016 described a patient that underwent a robotic-assisted laparoscopic RP after previously undergoing a suprapubic prostatectomy [42]. No abnormal findings were discovered, and the patient had no symptoms of urinary incontinence or difficulty voiding post-operatively. Additionally, he had a PSA of <0.01 ng/mL. Although there were favorable outcomes in this case, the researchers recommend that this procedure be conducted by skillful surgeons with extensive experience. Apart from this case, the management of iPCa after SP is not well documented in the literature, and thus, it is not possible to make any conclusions on effective treatment regimens for these patients. It would appear likely that most would be recommended for either active surveillance or observation with those undergoing treatment proceeding with radiation.

Management options for iPCa include active surveillance, watchful waiting, and curative therapies, such as RP, hormonal therapy, and radiotherapy. Studies suggest that AS is a viable option for low-risk iPCa cases, while curative therapies can be considered for selected patients. An overview of the management strategies and outcomes observed in various studies are shown in Table 1.

**Table 1:** Outcomes after treatment of PCa in patients with prior history of BPH surgery. iPCa: incidental prostate cancer; PCa: prostate cancer; BPH: benign prostatic hyperplasia; GS: Gleason score; PSA: prostate-specific antigen; MRI: magnetic resonance imaging; HoLEP: Holmium laser enucleation of the prostate; TURP: transurethral resection of the prostate; EBRT: external beam radiation therapy; AS: active surveillance; RP: radical prostatectomy

Reference	Patient characteristics	Summary of treatment	Outcome
Capitanio et al. [22]	126 had iPCa diagnosed between 1995 and 2007 at a single institution	Radical retropubic prostatectomy	5-year and 10-year biochemical recurrence-free survival rates were 92% and 87%, respectively, and the rates did not differ between T1a and T1b patients
Elkoushy et al. [25]	70/1242 (5.6%) had IPCa after HoLEP between 1998 and 2014 at a single institution	5 patients opted for chemotherapy/hormone therapy, 2 patients opted for EBRT, and 54 patients opted for AS	Kaplan-Meier survival analysis demonstrated an overall survival of 72.8% at 5 years and 63.5% at 10 years for patients with coexisting PCa at the time of HoLEP
Tombal et al. [26]	182/1648 (11%) had IPCa between 1985 and 1997 at a single institution. 125 had prior TURP and 56 had prior SP	66 patients elected for AS	4/49 T1a patients had biological progression within a mean follow-up of 73 months. 5/17 T1b patients had biological progression in a mean follow-up of 17 months. 5 years the cumulative biological recurrence-free survival probability was 93% for T1a and 70% for T1b.
Chung et al. [30]	A total of 95 patients were diagnosed with iPCa at a single institution between June 2006 and December 2016	RP	Following RP, 67 exhibited residual tumor and 28 did not (pT0). When dividing the two groups based on residual tumor, there were significant differences in PSA after BPH surgery, PSA density before and after BPH, and suspicious lesions in MRI. Among 67 RP specimens, 44 exhibited GS 6, and 16 exhibited GS 7. And 7 exhibited GS ≥8. Pathologic stage ≥T3 was recorded in 10 cases, the extracapsular extension was present in 10 cases, and surgical margins were involved in 4 cases. Invaded seminal vesicles were observed in 1 case. Perineural invasion was reported in 1 case. During the follow-up period, biochemical recurrence was not observed in pT0 patients; in the residual PCa group, BCR was observed in 11 cases. There were no cancer-specific deaths during the observation period. Kaplan-Meier curves showed a significant increase in biochemical recurrence-free survival in the pT0 group. In these analyses, GS ≥8 and pathologic T stage ≥T3 were independent prognostic factors for biochemical recurrence. In contrast, PSA, PSA density, and T1a or T1b were not statistically different. Patients with PSA density ≤0.08 after BPH surgery and with invisible cancer lesions on MRI should be considered for active surveillance
Tominaga et al. [31]	25/418 (6.0%) had IPCa after HoLEP between 2008 and 2016 at a single institution	2 underwent RP, 1 underwent radiotherapy, and 2 underwent hormone therapy	All patients that underwent cancer treatment or watchful waiting have survived without cancer progression (mean follow-up period 30.4 and 34.7 months, respectively); 5-year overall survival and progression-free survival rates are 100%
Lee et al. [32]	156 had IPCa detected after TURP between 2001 and 2012 at a single institution	46 elected for AS, 67 underwent RP, 34 received hormone therapy, 4 received radiotherapy, and 5 chose watchful waiting	During follow-up, no patients died due to prostate cancer
Hagmann et al. [33]	124/391 (32%) had IPCa after TURP between December 1999 and December 2020 at our institution	AS	The median follow-up was 46 months. Cancer-specific survival was 99.7% and overall survival was 92.3%
Menard et al. [34]	16/46 (35%) had IPCa after TURP between May 1998 and January 2005 at a single institution	RP	5-year survival rate without biochemical progression did not show a statistically significant difference between patients that previously had a TURP and patients that did not
Jaffe et al. [35]	119 had a history of TURP between January 1998 and December 2006 at a single institution	Laparoscopic RP	Mean estimated blood loss, transfusion rate, pathological prostate volume, and reoperation rate were statistically similar between patients that had a prior history of TURP and those that did not. Positive margins were seen in 21.8% and 12.6% of the patients with and without transurethral prostate resection, respectively. A total of 64 complications were seen in patients with a history of transurethral prostate resection compared to 34 in those without such a history
Abedali et al. [36]	27 had prior HoLEP at a single institution	Robot-assisted RP	Operative times were significantly longer with higher bladder-neck reconstruction rates and similarly low complication rates in patients with prior HoLEP. Biochemical recurrence was relatively low in the HoLEP group and identical to patients without a history of HoLEP. Continence at the last follow-up was not statistically significant between groups. Erectile function recovery was generally poor in the post-HoLEP cohort
Kretschmer et al. [37]	A total of 95 patients had a history of HoLEP between 2011 and 2019 at two institutions	RP	No significant impact of previous HoLEP on positive surgical margin rate and biochemical recurrence-free survival. Patients with a history of HoLEP had increased 1-year urinary incontinence rates after RP. Previous HoLEP did not hamper 1-year erectile function recovery
Guilhen et al. [39]	59 had a history of TURP between November 2008 and February 2013 at a single institution	EBRT	Five-year biochemical disease-free survival was 75% and 5-year clinical relapse-free survival was 84%. During the follow-up, 10 patients had a biochemical recurrence, 7 patients had loco-regional progression, 3 patients had metastatic progression
Devisetty et al. [40]	71 had a history of TURP between 1988 and 2005 at a single institution	EBRT	Compared to men without a prior TURP, TURP patients had a lower rate of freedom from late grade 3 genitourinary toxicity or higher after EBRT. Although, by the last follow-up, maximal GU toxicity tended to resolve and there was no worsening of urinary symptom scores
Tsui et al. [42]	1 patient with previous suprapubic SP	Robot-assisted laparoscopic prostatectomy	A voiding cystogram was performed 10 days post-op and did not demonstrate any extravasation or abnormal findings. On 9 weeks post-op visit, he was noted to be continent, with minimal difficulty voiding, and had a PSA of <0.01 ng/mL
Suardi et al. [43]	30 patients were diagnosed with IPCa between January 2004 and March 2006, 9 had prior TURP, 11 had prior HoLEP, and 10 had prior SP	Nerve-sparing robotic RP	At the last follow-up evaluation, no statistical differences were found among the groups in terms of urinary continence between patients with prior HoLEP, TURP, or SP after nerve-sparing RP. Two patients in each group (13.3%) developed an anastomotic stricture. The rate of positive surgical margins was significantly lower in the HoLEP group. Nerve-sparing RP is a feasible procedure in patients diagnosed with PCa who previously underwent HoLEP for BPE, although it may require higher surgical skills than in patients that never had previous prostate surgery
Lecumberri et al. [44]	80 patients were diagnosed with IPCa between 1980 and 2005 at a single institution	34 were treated with hormone therapy, 4 with RP, 3 with radiotherapy, and 39 elected for AS	Median survival was 8.87 years. Cancer-specific survival was 16 years in the low-risk group (Gleason score less than 6) and 6 years in the high-risk group (Gleason score greater than 7), with no differences by treatment. Hormone therapy was not beneficial for these patients

## Conclusions

Detection rates of iPCa differ greatly between studies and types of BPH surgery performed. It is inconclusive based on current literature as to whether HoLEP, TURP, or SP yields a greater rate of detection of iPCa. Factors that seem to contribute to a greater likelihood of iPCa detection include those such as PSA, prostate volume, and patient age. Though classically, it has been regarded that T1b PCa should be treated more aggressively than T1a PCa, current research suggests that the topic is much more nuanced. It is rare for patients that have iPCa to present with Gleason score of >2 or PSA >10. As a result, much of the current literature favors watchful waiting for patients diagnosed with T1a/b PCa. It is advised that patients with low to favorable intermediate-risk prostate cancer undergo post-operative PSA checks and MRI imaging before electing to choose between observation, surveillance without confirmatory biopsy, immediate confirmatory biopsy, or active treatment. Additionally, the literature suggests that expanding the current tumor, node, metastasis (TNM) staging classification to more than just two subdivisions of greater than or less than 5% cancerous tissue obtained could improve prognosis and management decisions in patients with early-stage disease by more clearly delineating what degrees of infiltration should raise the concern of further cancer progression and a subsequent need for treatment.

Questions of how to manage and treat patients with higher-risk iPCa continue to remain. Additionally, the technical challenges of RP after a HoLEP or SP, which may influence surgeon and patient decision-making after diagnosis of high-risk iPCa, have yet to be explored. Because iPCa tends to present as low-grade cancers with a minimal likelihood of progressing to more advanced stages, recommendations for the management of these patients are not comprehensive. Our paper suggests that by expanding the classifications of iPCa and prioritizing PSA and MRI checks after the detection of iPCa, we can provide more tailored treatment options for our patients. As the frequency of these outlet procedures continues to increase, further studies are needed to establish definite management protocols for iPCa.
